# Birmingham Throat and Ear Hospital

**Published:** 1895-08-24

**Authors:** 


### BIRMINGHAM THROAT AND EAR HOSPITAL.

The new in-patient department of the Birmingham Throat and Ear Hospital was opened on August 12th by Lord Leigh, president of the institution. There are twelve beds at present, and it is hoped in future to extend the accommodation to thirty beds, in addition to the quarantine ward, which is so essential a part of a hospital for diseases of the throat.

